# Skull Base Osteomyelitis: A Case Report and Literature Review

**DOI:** 10.7759/cureus.88466

**Published:** 2025-07-21

**Authors:** Nathaniel Dusini, Gregory M Buchek

**Affiliations:** 1 Internal Medicine, Wright-Patterson Medical Center, Wright-Patterson Air Force Base, USA; 2 Internal Medicine, Wright State University Boonshoft School of Medicine, Dayton, USA; 3 Internal Medicine/Infectious Disease, Wright-Patterson Medical Center, Wright-Patterson Air Force Base, USA; 4 Internal Medicine/Infectious Disease, Wright State University Boonshoft School of Medicine, Dayton, USA

**Keywords:** chronic antimicrobial suppression, cranial nerve, osteomyelitis, outpatient parenteral antibiotic therapy, pseudomonas aeruginosa, skull base

## Abstract

Skull base osteomyelitis (SBO) is a rare but potentially life-threatening serious infection. It is most often caused by *Pseudomonas aeruginosa* and typically affects elderly people with diabetes or weakened immune systems. We present an 80-year-old man with diabetes who was diagnosed with SBO with left facial nerve involvement after initial misdiagnosis and inadequate response to treatment for otitis externa and Bell’s palsy. Imaging and transmastoid biopsy confirmed the diagnosis. Although prolonged antibiotic therapy suppressed the infection, the left facial nerve palsy remained unresolved. This case highlights the diagnostic challenges, the need for early imaging and biopsy, and the importance of tailored treatment and long-term follow-up to prevent irreversible complications.

## Introduction

Skull base osteomyelitis (SBO) is a severe and invasive infection that affects the temporal, sphenoid, or occipital bones, resulting from infection in surrounding tissues. It typically arises as a complication of necrotizing external otitis (NEO) or, less frequently, paranasal sinus infections. It can also be a complication of improperly treated otogenic or sinonasal infection in elderly patients with diabetes or immunocompromised patients [1]. Prior to infection of the bone, when only soft tissues are involved, it is known as NEO. As the disease advances, it may spread to the mastoid and temporal bones and, in more extensive cases, to the temporomandibular joint or the nasopharynx [2]. SBO usually affects individuals older than age 65 with diabetes as a major risk [3]. Other associated conditions include immunocompromised states like HIV and certain cancers [1]. *Pseudomonas aeruginosa* is the most common causative agent, responsible for 75%-95% of cases, followed by *Staphylococcus* species and other gram-negative bacteria [4]. Cases with a fungal etiology can typically be found in immunocompromised individuals without diabetes [2]. Individuals with SBO typically develop severe otalgia, headache, and otorrhea. In advancing infection, conductive hearing loss and cranial nerve palsies can also be present, with facial nerves being the most commonly affected [3]. Individuals often receive antibiotic treatment for refractory NEO for months prior to diagnosis [1]. The extension of the infection is typically seen and classified by radiographic findings via computed tomography (CT), magnetic resonance imaging (MRI), positron emission tomography (PET), and in some cases bone scan [2]. Bone biopsy is often obtained in individuals with NEO to rule out malignancy and may be helpful if initial cultures are negative. SBO may often be initially misdiagnosed as malignancy, resulting in extensive work-up before infectious syndrome is diagnosed [5]. Treatment consists of pathogen-directed antibiotics or antifungals ranging from six to eight weeks and several cases requiring months [3,5]. The decision to stop antibiotic therapy depends on stabilization or improvement in clinical symptoms, resolution of radiographic findings, and normalization of erythrocyte sedimentation rate (ESR) and/or C-reactive protein (CRP) [3]. To highlight the clinical course and diagnostic challenges of SBO, we present the case of an elderly diabetic patient with facial nerve palsy and delayed diagnosis, and we review current literature on diagnostic and therapeutic approaches.

This case was previously discussed as a case presentation at the 2025 annual DAGMEC research forum on March 27, 2025.

## Case presentation

An 80-year-old man with non-insulin-dependent type 2 diabetes and bilateral sensorineural hearing loss managed with hearing aids initially presented to his primary care provider with a one-week history of sudden-onset, constant left ear pain and aural fullness. Otitis externa (OE) was diagnosed based on left external auditory canal erythema on exam, and he was treated with ciprofloxacin otic drops. He returned to the emergency department (ED) one week later, showing sudden left facial nerve paralysis. He was diagnosed with Bell’s palsy with a normal external auditory canal on examination. A normal external auditory canal was noted on examination during the ED visit. One month after the initial OE diagnosis, he had an ongoing evaluation for presumed Bell’s palsy, receiving an oral steroid treatment and a course of valacyclovir and amoxicillin-clavulanate. Routine referral to neurology resulted in continued presumed diagnosis of Bell’s palsy, and another course of oral steroids was initiated. There was no report of fever, weight loss, or involvement of other cranial nerves throughout this process.

**Figure 1:**
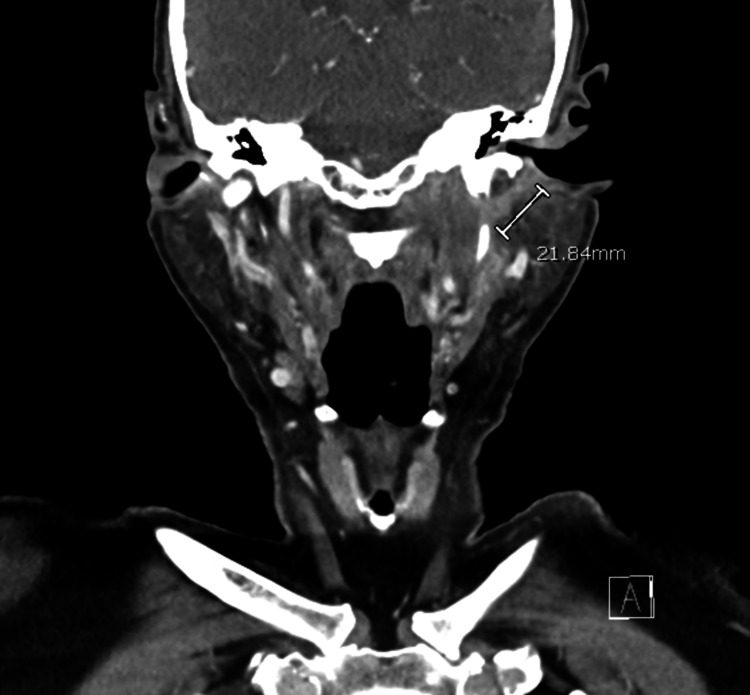
Computed tomography (CT) scan of neck with a coronal view showing hyperenhancing soft tissue along the superior medial deep portion of the left parotid space extending into the left parapharyngeal and carotid space.

**Figure 2:**
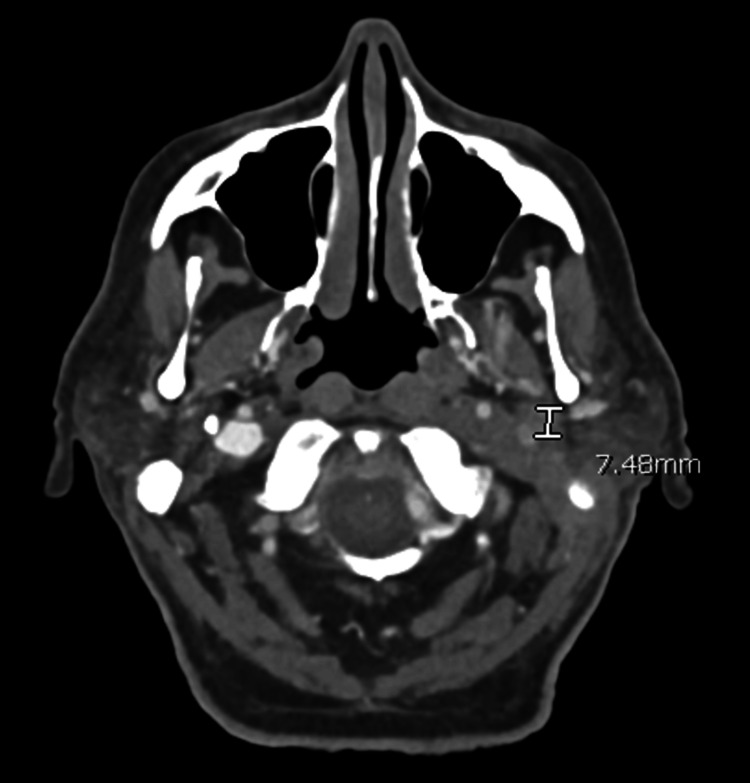
Computed tomography (CT) scan of the neck with a transverse view showing short-segment narrowing of the superior left internal cervical carotid artery.

**Figure 3:**
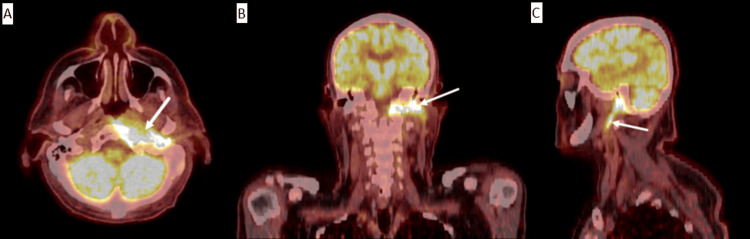
Positron emission tomography (PET) scan showing a fluorodeoxyglucose (FDG)-avid lesion (white arrow) involving posterior left nasopharynx, skull base, and tracking along the osseous course of the facial nerve with associated osseous erosion in transverse (A), coronal (B), and sagittal (C) views.

**Figure 4:**
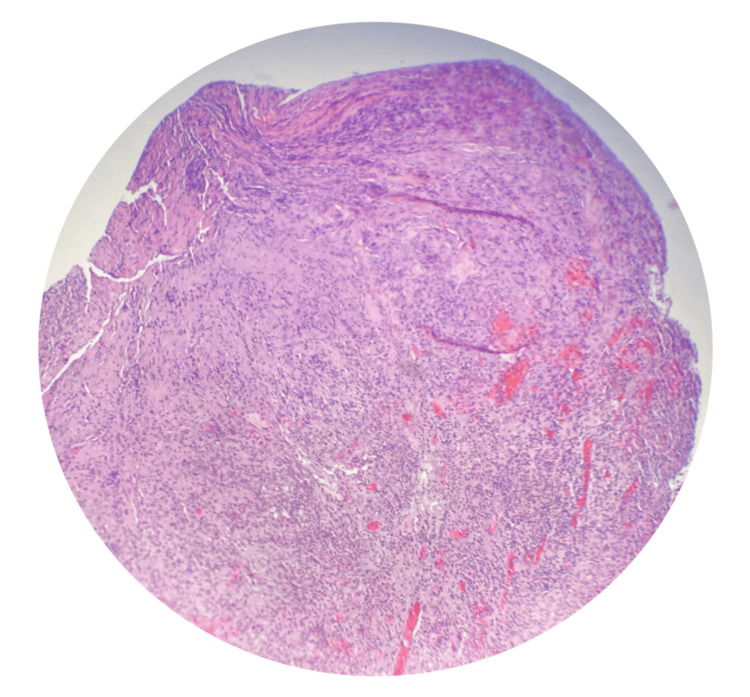
Histopathology of a biopsy obtained via left transmastoid biopsy, revealing granulation tissue and mixed inflammation.

He was first seen in the otolaryngology clinic two months after the initial OE diagnosis. It was noted that he had fluctuating symptoms of mild purulent otorrhea and otalgia. Left cranial nerve paralysis persisted, and so, CT neck soft tissue with contrast along with CT temporal bone without contrast was obtained. These demonstrated findings consistent with neoplasm involving the left facial nerve with extension into the left deep parotid, parapharyngeal, and carotid spaces (Figures 1, 2). A multidisciplinary tumor board recommended a PET scan, which revealed a fluorodeoxyglucose (FDG)-avid lesion involving the posterior left nasopharynx and skull base. The lesion followed the osseous course of the left facial nerve and showed associated bone erosion, raising concern for malignancy versus aggressive infection (Figure 3). Left transmastoid biopsy was immediately arranged, where an intraoperative exam revealed a grossly eroded and widened facial canal with obvious granulation and abnormal tissue. Histopathology was consistent with granulation tissue and mixed inflammation (Figure 4). *P. aeruginosa* was isolated from culture.

**Figure 5:**
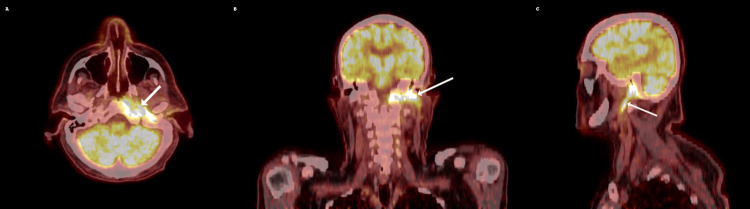
Repeat positron emission tomography (PET) scan 3 months after initial imaging showing stable fluorodeoxyglucose (FDG)-avid lesion (white arrow) involving posterior left nasopharynx, skull base, and tracking along the osseous course of the facial nerve with associated osseous erosion in transverse (A), coronal (B), and sagittal (C) views.

He was initiated on antipseudomonal therapy with intravenous piperacillin-tazobactam continuous infusion. Baseline laboratory tests were obtained (Table 1). These laboratory tests were significant for a slightly elevated white blood cell count, elevated CRP, and elevated ESR. The initial piperacillin-tazobactam duration was six weeks, but was extended to three months given good tolerance. Total resolution of otorrhea, resulting in the ability to once again use both hearing aids, was achieved one month into intravenous therapy. ESR and CRP normalized over the period of intravenous antibiotic therapy. Left facial nerve palsy remained stable but unimproved, with assessment by otolaryngology that there would be no recovery of function. A PET/CT scan was obtained at the completion of intravenous antibiotics, showing decreased metabolic activity at the left mastoid temporal bone region, but stable FDG avidity of the petrous apex of the skull base. Due to ongoing radiographic signs of disease activity and concern for possible relapse or progression to complications such as septic thrombophlebitis of the internal jugular vein, indefinite oral antipseudomonal suppression with ciprofloxacin was started (Figure 5).

**Table 1:** Comparative laboratory results: initial presentation vs. follow-up values. This table presents a comparison of baseline and follow-up laboratory test results for a patient, alongside the standard reference ranges for each parameter. The values include hematologic indices (e.g., WBC, RBC, and hemoglobin), inflammatory markers (CRP, ESR), electrolytes (sodium, potassium, etc.), renal function markers (creatinine, eGFR, and BUN), and other biochemical parameters. Notable changes between baseline and follow-up include normalization of WBC, a significant drop in platelet count, reduction in CRP but not ESR, and improvement in kidney function (eGFR). These data aid in tracking disease progression, response to therapy, and overall clinical improvement or deterioration. WBC: white blood cell; RBC: red blood cell; MCV: mean corpuscular volume; MCH: mean corpuscular hemoglobin; MCHC: mean corpuscular hemoglobin concentration; RDW: red blood cell distribution width; MPV: mean platelet volume; AGAP: anion gap; BUN: blood urea nitrogen; eGFR: estimated glomerular filtration rate; ESR: erythrocyte sedimentation rate; CRP: C-reactive protein

Lab name	Baseline lab value	Follow-up lab value	Reference range
WBC	11.9 × 10^3^/µL	8.0 × 10^3^/µL	4.6-9.4
RBC	4.57 × 10^6^/µL	4.56 × 10^6^/µL	4.40-5.90
Hemoglobin	12.3 g/dL	12.6 g/dL	13.0-18.0
Hematocrit	38.30%	39.30%	40.0-52.0
MCV	83.8 fL	86.3 fL	81.0-95.0
MCH	27.0 pg	27.6 pg	26.0-33.0
MCHC	32.2 g/dL	32.0 g/dL	32.0-36.0
RDW	14.70%	17.70%	11.5-14.5
Platelets	622 × 10^3^/µL	337 × 10^3^/µL	140-420
MPV	7.0 fL	7.8 fL	7.1-10.5
Neutrophil % auto	83.40%	65.80%	49.0-71.0
Lymphocyte % auto	9.50%	22.30%	20.0-40.0
Monocyte % auto	4.80%	5.40%	2.0-11.0
Eosinophil % auto	1.10%	5.10%	0.0-4.0
Basophil % auto	1.20%	1.40%	0.0-1.0
Lymph absolute	1.1 × 10^3^/µL	1.8 × 10^3^/µL	0.8-4.5
Mono absolute	0.6 × 10^3^/µL	0.4 × 10^3^/µL	0.1-1.3
ESR	38 mm/hr	84 mm/hr	0-15
CRP	7.32 mg/dL	2.59 mg/dL	≤0.50
Sodium	137 mmol/L	138 mmol/L	137-145
Potassium	3.80 mmol/L	3.90 mmol/L	3.50-5.10
Chloride	105 mmol/L	107 mmol/L	98-107
CO_2_	22 mmol/L	26 mmol/L	22-30
AGAP	10 mmol/L	5 mmol/L	6-18
BUN	24 mg/dL	12 mg/dL	9-20
Creatinine	1.50 mg/dL	1.10 mg/dL	0.66-1.25
eGFR	47 mL/min	68 mL/min	≥60
Glucose	115 mg/dL	184 mg/dL	74-106
Calcium	8.8 mg/dL	9.2 mg/dL	8.4-10.2
Albumin	3.60 g/dL	3.10 g/dL	3.50-5.00
Phosphorus	3.2 mg/dL	2.7 mg/dL	2.5-4.5

## Discussion

SBO is an uncommon but potentially life-threatening infection, often arising from NEO, particularly in elderly patients with diabetes or immunocompromised states [1]. This case illustrates a characteristic but diagnostically difficult progression of SBO, starting with presumed OE and developing into facial nerve palsy. The latter was initially misdiagnosed as Bell’s palsy, which delayed appropriate imaging and targeted treatment. Diagnostic delays are common in SBO, with multiple case series noting misdiagnosis as Bell’s palsy or chronic otitis, contributing to prolonged symptom duration before appropriate imaging is obtained [6,7]. Persistent symptoms despite standard therapies for Bell’s palsy and OE highlight the diagnostic ambiguity in early stages and the importance of maintaining a high index of suspicion when cranial nerve deficits are present.

Imaging modalities were vital for defining the extent of disease, with CT and MRI providing structural and soft tissue details, respectively, while FDG-PET/CT offered metabolic insights essential for distinguishing infection from malignancy and monitoring treatment response. CT identified osseous changes and soft tissue involvement. A PET scan further delineated metabolically active areas, helping to differentiate SBO from neoplastic processes. The involvement of deep parotid, parapharyngeal, and carotid spaces, as well as FDG-avid lesions along the petrous apex, emphasized the extent and complexity of this infection, given proximity to cranial nerves and other skull base structures. While CT is often the first-line imaging modality to assess bone erosion, MRI provides superior soft tissue contrast and is more sensitive in detecting early marrow changes and cranial nerve involvement [8]. The role of FDG-PET/CT is emerging, particularly in differentiating infection from malignancy and in assessing treatment response [4,9,10]. Biopsy helped distinguish SBO from malignancy. Histopathology showed granulation tissue with mixed inflammation and culture isolation of *P. aeruginosa*, consistent with literature citing this pathogen as the leading organism in 75%-95% of cases [3]. This reinforces the diagnostic value of histopathology in ambiguous imaging cases. Although *P. aeruginosa* remains the most commonly isolated pathogen, fungal organisms such as *Aspergillus* spp. and *Candida* spp. have increasingly been reported, especially in immunocompromised patients or when empiric therapy fails [3,11].

The result of favorable biochemical and clinical response to prolonged intravenous piperacillin-tazobactam, followed by indefinite oral ciprofloxacin due to radiographic persistence, aligns with current practices that advocate for extended and tailored therapy [5]. The case also illustrates the challenge of treatment duration and monitoring. Despite normalization of ESR and CRP, ongoing FDG uptake at the petrous apex raised suspicion of subclinical infection and led to the continuation of suppressive therapy. This emphasizes the limited reliability of inflammatory markers alone and highlights the potential usefulness of PET/CT in guiding therapy duration and anticipating complications such as septic thrombophlebitis. The decision to continue suppressive oral therapy reflects a risk-mitigating strategy. No consensus exists on treatment duration, with reports ranging from six weeks to over six months depending on radiologic and clinical response [1,4-6,12]. Future research could focus on defining PET/CT thresholds for treatment discontinuation [9,12].

In this case, facial nerve palsy persisted despite infection suppression, likely indicating irreversible nerve damage caused by prolonged inflammation, ischemia, and osteolysis-a common and often irreversible complication of delayed SBO treatment [5]. Facial nerve palsy is the most commonly involved cranial neuropathy in SBO and often portends a more severe disease course. Studies suggest variable recovery, with persistent deficits linked to delayed diagnosis or extensive bone involvement [7,13]. This underscores the need for early diagnosis and intervention to preserve neurological function. Furthermore, sensorineural hearing loss and reliance on hearing aids in this case added complexity to symptom evaluation and underscored the importance of audiologic function in quality of life for this population.

Several key principles in SBO management were reinforced in this case: early imaging and biopsy in persistent or atypical otologic symptoms, long-term antimicrobial therapy with close clinical and radiographic monitoring, and the need for individualized care in older adults with comorbidities. As diagnostic tools like 18F-FDG-PET/CT become more widely adopted [4], their role in monitoring disease activity post-treatment warrants further study. Likewise, the development of clear criteria for treatment duration and therapeutic endpoints remains an unmet need. Moreover, the absence of validated criteria for defining microbiological cure or radiologic resolution in SBO remains a significant gap in clinical practice, often leading to prolonged empiric treatment and variability in care protocols.

## Conclusions

We discussed SBO complicated by left facial nerve palsy in an 80-year-old male patient with a history of non-insulin-dependent type 2 diabetes. Prolonged intravenous pathogen-directed therapy against *P. aeruginosa* preceded by delayed radiographic, histologic, and culture diagnosis highlighted the complexity of this rare condition. Despite the resolution of otorrhea and normalization of inflammatory markers, this case emphasized irreversible complications, such as cranial nerve dysfunction, and the need for chronic suppressive antibiotics based on PET/CT monitoring showing persistent FDG avidity despite clinical improvement. Future research should focus on refining diagnostic criteria, optimizing the duration of therapy, and developing strategies to minimize long-term complications with an emphasis on improving outcomes.
